# First Report of Intervertebral Disc Calcification in a Black Child in Sub‐Saharan Africa

**DOI:** 10.1002/ccr3.71180

**Published:** 2025-10-07

**Authors:** Yamyelle Enselme Zongo, Ismael Ayouba Tinni, Hamiidah Hunsu‐Horhon Guingani, Yannick Laurent Tchenadoyo Bayala, Aboubakar Ouedraogo, Fulgence Kabore, Wendlassida Stéphanie Joelle Zabsonré/Tiendrebeogo, Dieu‐Donné Ouedraogo

**Affiliations:** ^1^ Rheumatology Department Bogodogo University Hospital of Ouagadougou Ouagadougou Burkina Faso; ^2^ Joseph KI‐ZERBO University of Ouagadougou Ouagadougou Burkina Faso

**Keywords:** Burkina Faso, calcifications, child, intervertebral disc

## Abstract

We report an intervertebral disc calcification in a Black African child who had consulted us for neck pain. The diagnosis of C2–C3 intervertebral disc calcification was made on a standard X‐ray and CT scan. Treatment was exclusively conservative. Intervertebral disc calcifications in children are rare and benign, requiring long‐term monitoring.

## Graphical abstract

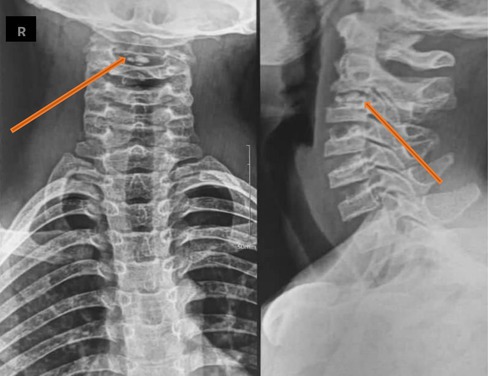

## 1 Introduction

First described in 1924 by Baron in a 12‐year‐old boy who presented with back pain, fever, and progressive thoracic scoliosis, intervertebral disc calcification was identified on imaging, without any evidence of associated bone involvement [1]. Intervertebral disc calcifications in children are a relatively rare condition, the exact frequency of which is not known [2]. Disc calcifications consist of a soft paste containing calcium hydroxyapatite crystals. They affect the nucleus pulposus and, more rarely, the entire vertebral disc [3]. The etiology of intervertebral disc calcifications is not yet known. The clinical manifestations range from a spinal pain crisis to a spinal cord or radicular compression syndrome that may require emergency decompression surgery, or a completely asymptomatic picture [4]. To date, no predisposing factors to symptomatic episodes have been definitively identified. We report the case of a Black African child who presented with acute neck pain and was diagnosed with intervertebral disc calcification. This case is of particular interest due to the rarity of the condition, especially in African populations, and the diagnostic challenges it poses. Through this report and a review of the literature, we aim to contribute to a better understanding of the clinical and radiological features of this uncommon pediatric entity.

## 2 Case Presentation/Examination

The case involved an 8‐year‐old male child who had received all vaccines included in the Expanded Programme on Immunization and had no relevant personal or family medical history. There was no history of trauma. He presented with acute mechanical neck pain that began after a long coach journey and had been evolving for one month in an afebrile context, with no alteration in general condition. There were no signs of otorhinolaryngology, stomatology, or other infectious sites.

The patient was classified as World Health Organization clinical stage 1 and had stable hemodynamic parameters. Musculoskeletal examination revealed abnormal cervical posture, pain on palpation of the cervical spinous processes, and paravertebral muscle contracture. Cervical spine mobilization was painful but without signs of stiffness (chin‐to‐sternum distance: 0 cm; occiput‐to‐wall distance: 0 cm). Examination of the remaining spinal segments and peripheral joints was unremarkable. Neurological examination showed normal consciousness, with no motor or sensory deficits. The remainder of the neurological and systemic examinations was also within normal limits.

## 3 Methods (Differential Diagnosis, Investigations, and Treatment)

**FIGURE 1:**
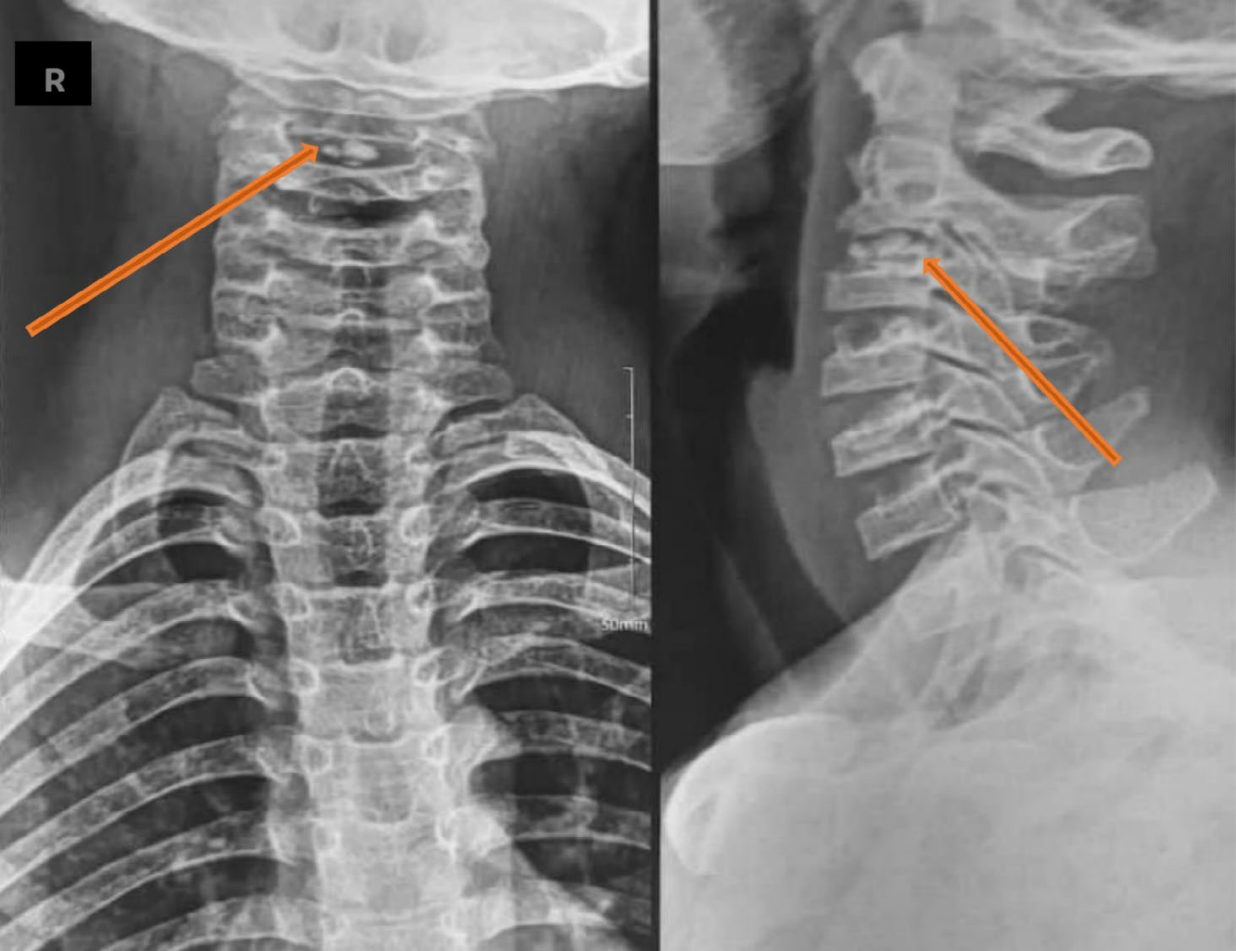
Standard X‐ray of the cervical spine from the front and in profile. Blurred appearance of the C2–C3 and C3–C4 vertebral endplates and calcification (orange arrow) in the intervertebral disc of C2–C3.

**FIGURE 2:**
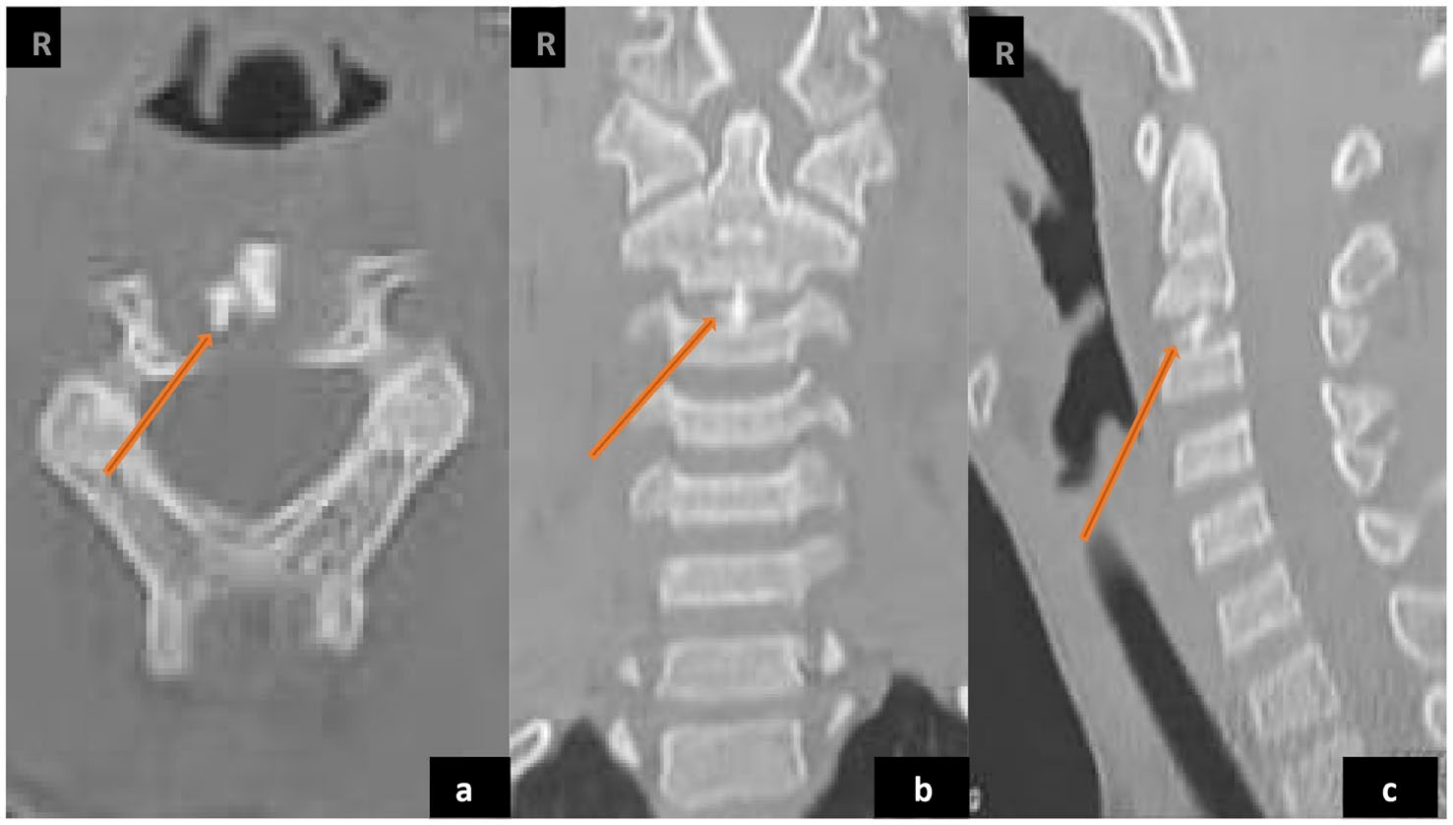
Cervical spine scan. Calcification (orange arrow) in the intervertebral disc of C2–C3 in axial (a), frontal (b), and sagittal (c) sections.

An initial diagnosis of torticollis was made, and local treatment with a non‐steroidal anti‐inflammatory ointment combined with physiotherapy sessions was initiated, but without clinical improvement. Due to the persistence of pain, standard anteroposterior and lateral radiographs of the cervical spine were performed. The radiographs revealed a blurred appearance of the C2–C3 and C3–C4 vertebral endplates, along with a calcified opacity within the C2–C3 intervertebral disc (Figure 1). To further investigate, a cervical CT scan was carried out, which confirmed the presence of discal calcification at the C2–C3 level, without any evidence of mass effect on the spinal cord (Figure 2). The diagnosis of intervertebral disc calcification was thus confirmed.

The complete blood count revealed a white blood cell count of 5320/μL, hemoglobin level of 13.6 g/dL, and a platelet count of 320,000/μL. Ferritin was low at 48.2 ng/mL, and parathyroid hormone was 12.9 pg/mL. Hemoglobin electrophoresis showed a hemoglobin A level of 96.6%. The remaining laboratory investigations were within normal limits: C‐reactive protein at 2.29 mg/L, serum iron at 8.92 μmol/L, alkaline phosphatase at 233 IU/L, blood calcium at 2.46 mmol/L, phosphorus at 1.70 mmol/L, and total 25‐hydroxyvitamin D (D2 + D3) at 30 ng/mL.

The patient was started on colchicine 1 mg, 0.5 mg twice daily in combination with a muscle relaxant (baclofen 10 mg) twice daily for 10 days. Rest was recommended, along with the use of a soft cervical collar.

## 4 Conclusion and Results (Outcome and Follow‐Up)

**FIGURE 3:**
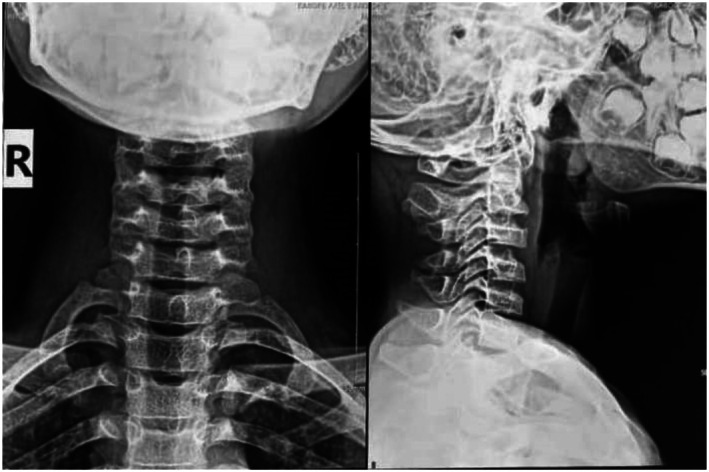
Standard x‐ray of the cervical spine, frontal, and lateral views. Calcification has disappeared.

The clinical course was marked by a gradual improvement in pain over three weeks, allowing the patient to resume daily activities. At a follow‐up visit 5 months later, the patient reported no neck pain. A follow‐up cervical spine radiograph (Figure 3) showed complete resolution of the previously observed calcification.

Intervertebral disc calcifications in children are rare and benign. The etiology is unknown. They should be considered in the presence of any unexplained spinal pain symptoms in children. The diagnosis is made by radiography. Treatment is conservative and combines analgesics, non‐steroidal anti‐inflammatories, muscle relaxants, reduced physical activity, and the wearing of a soft collar. Long‐term monitoring of these children is necessary.

## 5 Discussion

More than 300 cases have been reported in the literature to date. The exact frequency is not known. The cases reported concern exclusively Caucasian children [4]. To our knowledge, this is the first report of intervertebral disc calcification in a Black child in sub‐Saharan Africa. The absence of reported cases in Black African children may be explained by several factors. Intervertebral disc calcifications are asymptomatic in 15% of cases [3]. There is also a lack of awareness of the disease among nursing staff. Limited access to care and imaging tests, particularly CT scans and magnetic resonance imaging (MRI), although standard X‐rays are diagnostic in the typical clinical form [3].

Epidemiologically, the average age of onset is 8 years, with extremes ranging from 7 days to 20 years. There is a slight male predominance [2, 4]. To date, the cause of intervertebral disc calcifications is unknown. However, several factors have been incriminated, such as trauma, infection, autoimmune diseases, hypervitaminosis D, chondrocalcinosis, hyperparathyroidism, and hemochromatosis [5, 6]. In our case, no cause was found. The work‐up for infection was unremarkable. There was no evidence of autoimmune disease. Vitamin D, iron, and calcium levels were normal. On the other hand, it should be noted that in our patient, the pain had occurred on returning from a coach journey, which can lead to microtrauma to the spine.

Clinically, the most common presentation is a painful syndrome with stiffness (torticollis) accompanied by moderate hyperthermia and a biological inflammatory syndrome, which may suggest spondylodiscitis [3]. In a review of the literature, the most frequent clinical symptoms was muscle pain in 46 patients (71.9%), followed by sensorimotor disorders in 13 patients (20.3%) and fever in nine patients (13.8%). Torticollis or scoliosis was observed in 15 patients (23.4%), and ten patients (15.4%) were asymptomatic [4]. In our patient, the damage was localized in the C2–C3 space. All segments of the spine can be affected, with a clear predominance in the cervical region. The most affected space is C6–C7 [7]. Damage to the C2–C3 space has been reported in China [8].

Biologically, there was a drop in ferritin in our patient. In the literature, biological tests frequently show moderate hyperleukocytosis, increased sedimentation rate, and hyperproteinorachia. In the Chinese series, the authors point out that inflammatory signs are rare [8, 9].

In terms of diagnosis, standard X‐rays are usually sufficient and show round or oval calcifications involving the nucleus pulposus [6, 9, 10]. In addition, radiological signs of disc protrusions may be found, most often in the lower cervical region or in the upper thoracic region in 38% of cases. Clinical manifestations are constant, with a characteristic attack of spinal pain. In addition, in cases of posterior protrusion, neurological manifestations such as spinal cord or radicular compression may be encountered. In the case of anterior expulsion of the disc calcification, swallowing problems may occur [11, 12]. Computed tomography and MRI can be used to determine the extent of intra‐canal penetration, which averaged 17% of the sagittal diameter (0%–64%) [8]. In our patient, the diagnosis was made radiographically and confirmed by a CT scan.

When diagnosing torticollis or back pain, it is important to rule out certain differential diagnoses, the most important of which is infectious spondylodiscitis, which can present clinically as intervertebral disc calcification. But in the case of spondylodiscitis, the pain is much more severe. On imaging, in spondylodiscitis, there is pinching of the intervertebral disc and erosion of the adjacent vertebral endplates without any visible calcification [2]. Chronic inflammatory rheumatism, particularly spondylarthritis, should also be considered. Radiological examination can rule out the diagnosis of spondylarthritis due to the absence of vertebral squaring, syndesmophytes, and sacroiliitis.

Complications can occur, such as anterior hernias with erosion of the anterosuperior angle of the underlying vertebra and sometimes esophageal compression and dysphagia, foraminal hernias with radicular compression, or posterior hernias with subligamentary intracanal migration without compression [3, 11, 12].

Treatment is conservative. It combines analgesics and non‐steroidal anti‐inflammatory drugs, muscle relaxants, reduced physical activity, and the use of a soft collar or moderate traction until symptoms resolve [8, 13]. Our patient was treated with colchicine. Previous studies had not used colchicine as a treatment. However, our patient had a favorable outcome after 3 weeks. Further studies will evaluate the efficacy of colchicine in this indication. It should also be noted that spontaneous resolution of calcification is possible, as 15% of cases are asymptomatic [3]. In 70% of cases, the pain attacks disappear within a month, and the calcifications disappear within 28 days to 6 months [12, 14, 15].

The prognosis for intervertebral disc calcifications in children is excellent. Symptoms regress within a few days to a few weeks after the start of symptomatic treatment. Neurological forms, particularly spinal cord compression, should be monitored and may require surgery, although this is rare. With this proviso, conservative treatment is recommended, and radicular or spinal compression by a calcified cervical disc in children should not be considered an absolute indication for surgery [8].

Any interpretation of our clinical case must take into account the following limitations: the lack of functional analysis at the time of radiography, the lack of biopsy of the calcification, and the relatively short follow‐up of the patient.

## Author Contributions

**Yamyelle Enselme Zongo:** conceptualization, methodology, supervision, validation, visualization, writing – original draft. **Ismael Ayouba Tinni:** conceptualization, methodology, supervision, validation, visualization, writing – original draft. **Hamiidah Hunsu‐Horhon Guingani:** visualization, writing – original draft. **Yannick Laurent Tchenadoyo Bayala:** visualization, writing – original draft. **Aboubakar Ouedraogo:** validation, visualization. **Fulgence Kabore:** validation, visualization. **Wendlassida Stéphanie Joelle Zabsonré/Tiendrebeogo:** supervision. **Dieu‐Donné Ouedraogo:** supervision.

## Consent

I confirm that the patient's written consent has been signed and obtained in accordance with the journal's patient consent policy from the patient's legal guardians and that I have added a patient consent statement asserting this at the bottom of the manuscript's title page. I will retain the original written consent form and provide it to the publisher if requested.

## Conflicts of Interest

The authors declare no conflicts of interest.

## Data Availability

The data that support the findings of this study are available from the corresponding author upon reasonable request.
